# Expression of calcium-buffering proteins in rat intrinsic laryngeal muscles

**DOI:** 10.14814/phy2.12409

**Published:** 2015-06-24

**Authors:** Renato Ferretti, Maria Julia Marques, Tejvir S Khurana, Humberto Santo Neto

**Affiliations:** 1 Departamento de Anatomia, Instituto de Biociencias de Botucatu, Universidade Estadual PaulistaBotucatu, São Paulo, Brazil; 2 Departamento de Biologia Estrutural e Funcional, Instituto de Biologia, Universidade Estadual de CampinasCampinas, São Paulo, Brazil; 3 Department of Physiology, Perelman School of Medicine and Pennsylvania Muscle Institute, University of PennsylvaniaPhiladelphia, Pennsylvania

**Keywords:** Calcium, laryngeal muscles, Ncx, Pmca1, Serca, store-operated calcium entry

## Abstract

Intrinsic laryngeal muscles (ILM) are highly specialized muscles involved in phonation and airway protection, with unique properties that allow them to perform extremely rapid contractions and to escape from damage in muscle dystrophy. Due to that, they may differ from limb muscles in several physiological aspects. Because a better ability to handle intracellular calcium has been suggested to explain ILM unique properties, we hypothesized that the profile of the proteins that regulate calcium levels in ILM is different from that in a limb muscle. Calcium-related proteins were analyzed in the ILM, cricothyroid (CT), and *tibialis anterior* (TA) muscles from male Sprague–Dawley rats (8 weeks of age) using quantitative PCR and western blotting. Higher expression of key Ca^2+^ regulatory proteins was detected in ILM compared to TA, such as the sarcoplasmic reticulum (SR) Ca^2+^-reuptake proteins (Sercas 1 and 2), the Na^+^/Ca^2+^ exchanger, phospholamban, and the Ca^2+^-binding protein calsequestrin. Parvalbumin, calmodulin and the ATPase, Ca^2+^-transporting, and plasma membrane 1 were also expressed at higher levels in ILM compared to TA. The store-operated calcium entry channel molecule was decreased in ILM compared to the limb muscle and the voltage-dependent L-type and ryanodine receptor were expressed at similar levels in ILM and TA. These results show that ILM have a calcium regulation system profile suggestive of a better ability to handle calcium changes in comparison to limb muscles, and this may provide a mechanistic insight for their unique pathophysiological properties.

## Introduction

The intrinsic laryngeal muscles (ILM) are highly specialized muscles involved in vital functions that include respiration, phonation, and airway protection (Hinrichsen and Dulhunty 1982; DelGaudio et al. 1995; Hoh 2005). These functions are possible due to ILM unique features that include specific fiber-type composition, high speed contraction, and resistance to fatigue (Hinrichsen and Dulhunty 1982; Hoh 2005). Their ability to precisely control airway caliber during phonation requires specific characteristics reflected in their mechanical properties, innervation pattern (Maranillo et al. 2005; Shinners et al. 2006; Xu et al. 2009), regenerative capacity, response to age (Kersing and Jennekens 2004; Goding et al. 2005; McLoon et al. 2007), and disease (Marques et al. 2007; Thomas et al. 2008; Smythe 2009). Within the ILM, the posterior cricoarytenoid (PCA) muscle abducts the vocal fold, whereas the thyroarytenoid and cricothyroid (CT) adducts and tenses the vocal fold to aid in phonation and airway protection during swallowing (Hinrichsen and Dulhunty 1982; Hoh 2005). An interesting property of the ILM, in comparison to limb muscles, is their differential involvement in neuromuscular disorders. While ILM are involved early in myasthenia gravis, amyotrophic lateral sclerosis with bulbar involvement, and mitochondrial myopathy (Debain et al. 1968; Mao et al. 2001), they are histologically spared in Duchenne muscle dystrophy (DMD), except for the CT muscle (Marques et al. 2007; Thomas et al. 2008; Smythe 2009).

The ILM play important roles in initiation of and recovery from acute respiratory distress syndrome (ARDS) and critical illnesses, with both swallowing and phonation disability (Nixon et al. 2010). Additionally, vocal muscle paralysis is an important and frequent condition seen in otolaryngology clinics, most frequently caused by lesions to the recurrent laryngeal nerve (Li et al. 2014), although idiopathic etiologies remain common causes of unilateral vocal fold immobility (Rosenthal et al. 2007). Possible mechanisms underlying this differential response of ILM to muscle diseases, compared to limb muscles, may be related to differences regarding their neuromuscular junction structure or satellite cell regenerative potential (Feng et al. 2012). In the *mdx* mouse model of DMD (Bulfield et al. 1984), we have suggested that ILM differ from the affected limb muscles in terms of their ability to handle changes in calcium due to differential levels of calcium-buffering proteins (Ferretti et al. 2009), similarly to what is observed in the extraocular muscles, which are also protected in dystrophy (Khurana et al. 1995; Porter et al. 1998).

In skeletal muscle fibers, intracellular calcium (Ca^2+^) plays an important role in regulating muscle force production, metabolism, and muscle gene expression (Berchtold et al. 2000; Chin 2010). The sarcoplasmic reticulum (SR) and T-tubule membrane proteins play central roles in calcium regulation. In excitation contraction coupling, depolarization of the plasma membrane leads to the release of Ca^2+^ from the SR through ryanodine receptors (RyRs), which are opened via a direct physical interaction with the dihydropyridine receptors (DHPR, CACNA1S), voltage sensors localized in the T-tubules (Melzer et al. 1995; Gailly 2002). At the end of stimulation, Ca^2+^ is pumped back into the SR by sarcoplasmic reticulum Ca^2+^ ATPases (SERCA), extruded from the cell by the Na^+^/Ca^2+^ exchanger (NCX) (Berridge et al. 2003) and buffered by the low-affinity, high capacity Ca^2+^-binding protein calsequestrin (CASQ) (Beard et al. 2004). Other proteins that also play a role in Ca^2+^ regulation include the T-system plasma membrane Ca^2+^-ATPase type I (PMCA1) and the store-operated calcium entry (SOCE), which uses the SR Ca^2+^ sensor Orai1 Ca^2+^ channel (Lang et al. 2012). In addition, cytosolic Ca^2+^-binding proteins, such as parvalbumin (PVALB), calmodulin (CaM), and regucalcin (RGN), can modify Ca^2+^ transients to ensure Ca^2+^ homeostasis (Gailly 2002; Berridge et al. 2003; Beard et al. 2004; Yamaguchi 2005; Lang et al. 2012).

In this study, we evaluated the profile of calcium regulation systems in ILM compared to limb muscle (*tibialis anterior*, TA), in adult rats, using qPCR and Western blotting. We were interested to see whether ILM shows a constitutive calcium-buffering proteins profile that could predict their better capacity to handle calcium changes in comparison to limb muscle and EOM. We found that Ca^2+^ reuptake-related proteins of the sarcoplasmic reticulum (*Serca1, Serca2, Pln, Casq1, and Casq2*) were expressed at higher levels in ILM in comparison to the limb muscle. ILM showed higher levels of *Orai1* in relation to TA and CaM was 17-fold increased in ILM compared to TA. A similar pattern of gene expression of Sercas 1 and 2 and calsequestrins was observed in the dystrophin-deficient *mdx* mice compared to the rat. These results provide a mechanistic insight into ILM physiological properties and response to pathophysiological conditions.

## Methods

### Animals

All animal experiments were performed in accordance with the ARVO Statement for the Use of Animals in Ophthalmic and Vision Research, using protocols approved by the Institutional Animal Care and Use Committee of the University of Pennsylvania School of Medicine (protocol #A3079-01) and by the Committee of the University of Campinas (protocol #1463-1). Male Sprague–Dawley rats (*n* = 20; 8 weeks of age), control C57BL/10ScSnJ (Ctrl, *n* = 10; 8 weeks of age), and *mdx* mice (C57BL/10ScSn-*Dmd*^*mdx*^/J; *n* = 10; 8 weeks of age) obtained from Jackson Laboratory were used in all experiments. The mice and rats were housed according to institutional guidelines and received food and water ad libitum during all experiments.

The muscles studied were the ILM (thyroarythenoid, posterior, and lateral cricoarythenoid), the cricothyroid (CT) muscle isolated from the other ILM and the *tibialis anterior* (TA) muscle from rats and dystrophic mice. The CT muscle was evaluated separately as it is affected in comparison to the other ILM, which are spared in dystrophin-deficient *mdx* mice (Marques et al. 2007; Thomas et al. 2008; Smythe 2009). We also analyzed the extraocular muscles (EOM: medial, lateral, superior, and inferior rectus), for further comparison of the mechanisms of calcium handling in very fast muscles (Zeiger et al. 2010).

### RNA isolation and SYBR Green-based qPCR

RNA isolation was performed using Trizol reagent (Ambion, Austin, TX) in combination with RNeasy Fibrous Tissue Mini Kit following the RNA protocol as indicated by the manufacturer (Qiagen, Valencia, CA). For SYBR Green qPCR up to 5 *μ*g of RNA was reverse transcribed using the Superscript II First Strand Synthesis kit using Oligo(dT) primers according to the manufacturer's instructions. Primers were designed using PrimerExpress 2.0 (Applied Biosystems, Foster City, CA) across exon boundaries (Table1). qPCR was run on a 7900HT ABI Prism real-time PCR instrument (Applied Biosystems). GAPDH served as reference gene. Fold change and statistical analysis was performed and *P* < 0.05 considered statistically significant.

**Table 1:** Primers designed using PrimerExpress 2.0 (Applied Biosystems) across exon boundaries

Accession number	Gene	Gene Symbol	Forward	Reverse
NM_017290	*Atp2a1*	*Serca1*	5′-GCTCGGAACTATCTGGAGGGA-3′	5′-GGCACAAGGGCTGGTTACTTC-3′
NM_058213.1	*Atp2a2*	*Serca2*	5′-GGGAGAACATCTGGCTCGTG-3′	5′-GCGGTTACTCCAGTATTG-3′
NM_053311	*Atp2b1*	*Pmca1*	5′-GTAGTGGCCGTGATTGTCGC-3′	5′-AGCGTGTCCATGATGAGGTTG-3′
L04684.1	*Cacna1s*	*Cacna1s*	5′-CCCCTGTCATGGCTAACCAA-3′	5′-GCCTGGGTTCTGAGGGAAGTC-3′
NM_031969	*Calmodulin 1*	*Calm*	5′-AATCCGTGAGGCATTCCGAG-3′	5′-TCTGTTAGCTTTTCCCCGAGG-3′
XM_001063867	*Calsequestrin1*	*Casq1*	5′-GACTTCCCACTGCTGGTCCC-3′	5′-TCCATATGCTGTCCGCATCC-3′
NM_017131	*Calsequestrin2*	*Casq2*	5′-GGAGCATCAAAGACCCACCC-3′	5′-TTCTCCGCAAATGCCACAAT-3′
NM_017008	*Gapdh*	*Gapdh*	5′-CCATGGAGAAGGCTGGGG-3′	5′-CAAAGTTGTCATGGAT-3′
NM_019268	*Ncx1/Slc8a1*	*Ncx*	5′-TTGTCGCTCTTGGAACCTCAG-3′	5′-GCTTCCGGTGACATTGCCTAT-3′
NM_022499	*Parvalbumin*	*Pvalb*	5′-CATTGAGGAGGATGAGCTGGG-3′	5′-CTTGTCTCCAGCAGCCATCAG-3′
NM_022707	*Phospholamban*	*Pln*	5′-GCTGAGCTCCCAGACTTCACA-3′	5′-TTGACAGCAGGCAGCCAAAC-3′
NM_031546	*Regucalcin*	*Rgn*	5′-ACGTGACATGTGCCAGGGAT-3′	5′-GCAATTCCTTTGACCCCAAGA-3′
XM_341818	*Ryanodine Receptor 1*	*RyR1*	5′-CAAGCGGAAGGTTCTGGACA-3′	5′-TGTGGGCTGTGATCTCCAGAG-3′

### Western blot analysis

Western blot analysis was performed using the NuPage System precast gels as described by the manufacturer (Invitrogen, Carlsbad, CA). Crude whole muscle homogenates were prepared using TNEC lysis buffer (50 mmol/L Tris-HCl pH 8, 150 mmol/L NaCl, 1% Igepal, 2 mmol/L EDTA) containing a complete protease inhibitor cocktail and PhosStop phosphatase inhibitors (Roche, Basel, Switzerland) (Ferretti et al. 2009). The protein concentration was determined using the DC assay (BioRad). Equal amounts (30 *μ*g) of samples were resolved on 4–12% Bis-Tris gels, transferred onto nitrocellulose membranes (Milipore, Billerica, MA) and probed with the following mouse or rabbit antibodies: anti-SERCA 1 (mouse monoclonal Serca1 ATPase, MA3-911, Affinity Bioreagents, Golden, CO); anti-SERCA2 (rabbit polyclonal Serca2 ATPase, ab3625, Abcam, Cambridge, MA); anti-Calsequestrin (recognizes Casq1 and Casq2; rabbit polyclonal, PA1-913, Affinity Bioreagents, Golden, CO); anti-Calmodulin (rabbit monoclonal, MA3-918, Affinity Bioreagents, Golden, CO); anti-CaMkIIB (mouse monoclonal, ab72604, Abcam, Cambridge, MA); anti-ORAI 1 (rabbit polyclonal, ProSci Incorporated, #4281); Glyceraldehyde-3-phosphate dehydrogenase (GAPDH, rabbit polyclonal, Santa Cruz Biotechnology, Santa Cruz, CA). Secondary goat-anti-mouse or goat-anti-rabbit antibodies were conjugated with horseradish peroxidase (Jackson Immuno Research, West Grove, PA). Protein bands were detected with a LAS-3000 Fuji imaging system (Fujifilm, Tokyo, Japan). Equal loading was confirmed after the transfer by Ponceau S staining (Sigma, St. Louis, MO). Bands were quantified densitometrically using Image J software (http://rsbweb.nih.gov/ij/ National Institutes of Health). Statistical analysis was carried out using Student's t-test, with *P* < 0.05 considered significant.

## Results

### Increased mRNA expression of Ca^2+^-handling proteins in rat ILM

We determined mRNA levels of genes involved in Ca^2+^ homeostasis in ILM's and TA muscles by qPCR. On the basis of a previous report (Zeiger et al. 2010), we separated the genes into five functionally distinct groups of Ca^2+^-related proteins (Table2). The first group contained genes of the sarcoplasmic, plasma membrane Ca^2+^ pumps, and the Na^+^/Ca^2+^ exchanger. The second group contained genes that act as regulators of the Serca pumps. The third group contained the main Ca^2+^-binding proteins of the SR. The fourth group of genes contained cytosolic Ca^2+^-binding proteins that buffer elevated intracellular Ca^2+^. The fifth group of genes examined included Ca^2+^ channels involved in EC coupling.

**Table 2:** Genes encoding Ca^2+^-handling proteins analyzed in intrinsic laryngeal muscles (ILM), cricothyroid (CT), and *tibialis anterior* (TA) muscles of adult rats. The mRNA levels of these genes were determined by SYBR Green qPCR. Sarcoplasmic reticulum (SR)

Protein category	Protein Function	Gene	ILM versus TA		CT versus TA		ILM versus CT	
			Fold-change	Statistical significance	Fold-change	Statistical significance	Fold-change	Statistical significance
Pumps	SR Ca2+-ATPase, fast twitch	*Sercal*	1.5 ± 0.43	n.s.	0.7 ± 0.29	<0.05	4.9 ± 1.35	<0.05
	SR Ca2+-ATPase, slow twitch	*Serca2*	1.5 ± 0.40	<0.05	1.2 ± 0.34	n.s.	3.1 ± 0.88	<0.05
	Ca2+-ATPase 1, plasma membrane	*Pmcal*	34.7 ± 14.9	<0.05	11.5 ± 2.75	<0.05	2.3 ± 0.53	n.s.
	Na+/Ca2+-exchange, plasma membrane	*Ncx*	19.8 ± 7.45	<0.05	5.2 ± 1.47	<0.05	3.5 ± 0.45	<0.05
Regulatory proteins	SR, regulates SERCA2	*Phospholamban*	9.6 ± 0.36	<0.05	4.9 ± 1.02	<0.05	2.2 ± 0.28	<0.05
	cytosol, regulates SERCA	*Regucalcin*	2.0 ± 0.55	n.s.	5.4 ± 1.1	<0.05	0.4 ± 0.50	<0.05
SR calcium binding proteins	SR, calcium binding protein, skeletal	*Calsequestrin 1*	1.0 ± 0.25	<0.05	1.2 ± 0.07	n.s.	0.9 ± 0.17	n.s.
	SR, calcium binding protein, skeletal	*Calsequestrin 2*	9.6 ± 2.61	<0.05	1.5 ± 0.25	n.s.	2.9 ± 0.65	<0.05
Cytosolic calcium binding proteins	Cytosol, calcium binding protein	*Parvalbumin*	1.8 ± 10.27	<0.05	0.8 ± 0.08	n.s.	2.1 ± 0.42	<0.05
	Calcium binding protein	*Calmodulin*	6.5 ± 1.41	<0.05	5.0 ± 1.00	<0.05	2.3 ± 0.74	n.s.
Calcium channels	Calcium channel subunit	*Cacna is*	0.4 ± 0.08	n.s.	1.1 ± 0.32	n.s.	2.6 ± 0.90	n.s.
	Calcium release channel, skeletal	*Ryanodine receptor I*	0.2 ± 0.68	n.s.	0.4 ± 0.04	n.s.	1.5 ± 0.37	n.s.

Mean fold changes ± SEM from three independent muscle samples, each sample containing muscles pooled from three or four different rats were calculated and analyzed using REST 2005. *P* < 0.05 was considered statistically significant.

**Figure 1:**
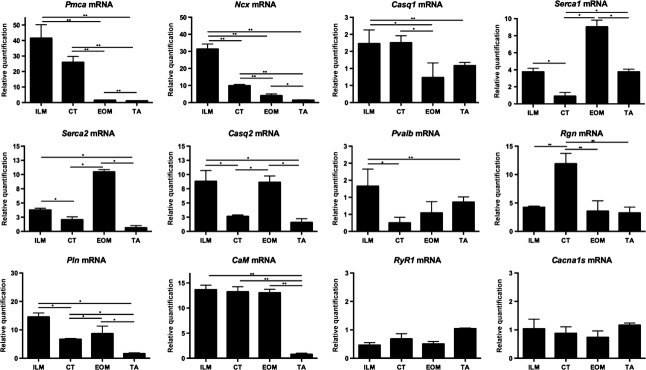
Relative mRNA levels of genes encoding Ca^2+^-handling proteins in intrinsic laryngeal muscles (ILM), cricothyroid (CT) muscle, extraocular muscle (EOM), and *tibialis anterior* (TA) muscle. The genes studied were Ca^2+-^ATPase 1, plasma membrane (*Pmca1*), Na^+^/Ca^2+-^exchange, plasma membrane (*Ncx*), Ca^2+^-ATPase slow twitch (Serca2), calsequestrin 2 (*Casq2*), phospholanbam (*Pln*), calmodulin (*CaM*), calsequestrin 1 (*Casq1*), Ca^2+^-ATPase, fast twitch (*Serca1*), parvalbumin (Pvalb), regucalcin (*Rgn*), ryanodine receptor 1 (*RyR1*), and calcium channel subunit (*Cacna1s*). The relative mRNA levels of genes encoding Ca^2+^-handling proteins were determined by SYBR Green qPCR in muscle tissue. Quantifications from three independent muscle samples, each sample containing muscles pooled from three or four different rats. Mean fold changes ± SEM of three independent samples each were calculated and statistically analyzed using REST 2005 and *P < 0.05, **P < 0.01 was considered statistically significant.

The mRNA of eight (Serca2, *Casq1, Casq2, Pln, Pvalb, CaM, Pmca1,* and *Ncx*) of the 12 genes quantified were expressed at significantly higher levels in ILM in comparison with TA (Fig.1 and Table2). The highest fold change was detected for the plasma membrane Ca^2+^pumps *Pmca1* and *Ncx* (up to 30-fold increase in ILM vs. TA). *Pln* and *Casq2* (about ninefold increase), followed by *CaM* presented a sixfold increase in ILM versus TA, (Fig.1 and Table2). *Pvalb* was increased to a lesser extent (1.8-fold increase in ILM compared to TA; Fig.1 and Table2). There were no differences in mRNA expression levels of *Rgn*, *Serca1*, *RyR1,* and *Cacna1s* in ILM compared to TA muscle (Fig.1 and Table2).

In the cricothyroid (CT) muscle, most of the quantified mRNAs (7 of 12; 58%) were significantly lower expressed in comparison with other ILM (Fig.1 and Table2), with main changes seen in *Pvalb* (twofold decrease in CT vs. ILM), followed by *Serca1* and *Casq2* (about 3.6-fold decrease in CT compared to ILM). *Rgn* was higher expressed in CT than in all the other muscles (Fig.1 and Table2). Conversely, the expression of most of the mRNAs in CT (6 of 12; 50%) was comparable to that observed in the limb muscle. *Rgn, Pln, Pmca1, Ncx,* and *CaM* were expressed significantly higher in CT than in TA.

The comparison of EOM with ILM showed that ILM display a similar profile of mRNA expression seen in EOM, for most of the proteins studied, except for Pmca1 and Ncx (higher in ILM than EOM) and Serca2 (higher in EOM than ILM).

### Increased protein levels of Ca^2+^-handling proteins in rat ILM

**Figure 2:**
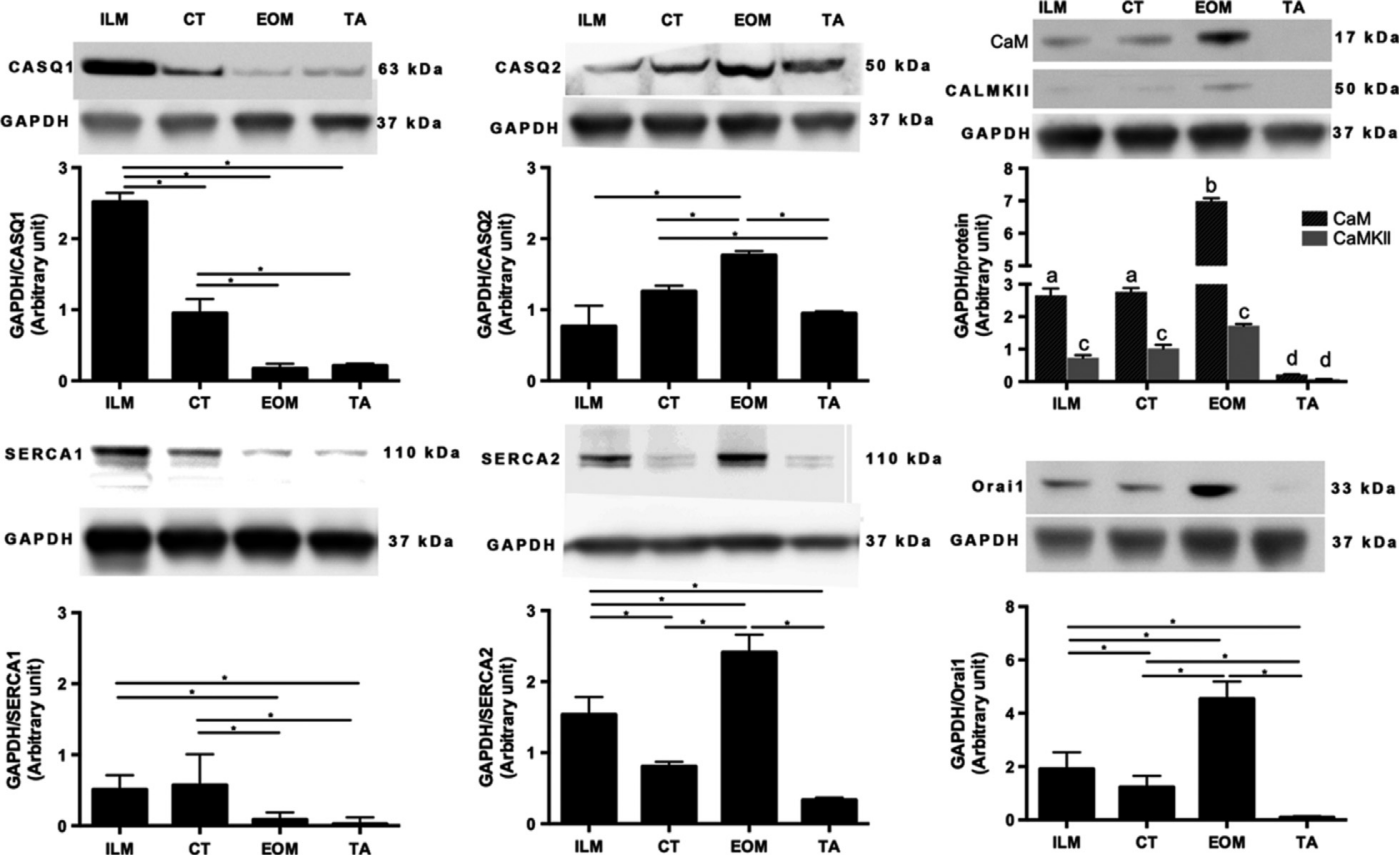
Protein levels of sarcoplasmic reticulum Ca^2+^-handling proteins in intrinsic laryngeal muscles (ILM), cricothyroid (CT) muscle, extraocular muscles (EOM), and *tibialis anterior* (TA) muscle. Western blot analysis showing relative abundance of indicated proteins: calsequestrins 1 and 2 (CASQ1 and CASQ2), Sercas 1 and 2 (SERCA1 and SERCA2), calmodulin (CaM), calmodulin kinase II (CaMKII), and Orai1. Glyceraldehyde-3-phosphate dehydrogenase (GAPDH) was used as a control for protein loading, Western blot transfer and nonspecific changes in protein levels. The molecular weight, expressed in kDa, for each protein is indicated. Quantifications from three independent muscle samples, each sample containing muscles pooled from three or four different rats. Asterisks and different letter combination indicate statistical significance (*P < 0.05 and ab, ac, ad, bc, or bd *P* < 0.05, respectively). In ILM, CASQ 1 was more abundant than CASQ2 compared with TA. SERCA1 was less than SERCA2 in ILM and higher compared with TA. CT and the other ILM showed similar levels of the proteins studied.

The relative expression levels of selected proteins involved in Ca^2+^ homeostasis were examined using western blots. Figure2 shows the levels of CASQ1 and CASQ2, SERCA1 and SERCA2, CaM, CaMKII, and Orai1. ILM presented higher levels of CASQ1, SERCA1, SERCA2, CaM, CaMKII, and Orai1 compared to TA (Fig.2). CASQ2 was higher in TA than in ILM. The levels of the proteins in CT were increased in relation to the levels observed in limb (CASQ1, CASQ2, SERCA1, CaM, CaMKII, and Orai1) and closer to the levels observed in ILM (CASQ2, SERCA1, and CaM and CaMKII).

### mRNA expression of calsequestrin and Serca in the *mdx* mice

**Figure 3:**
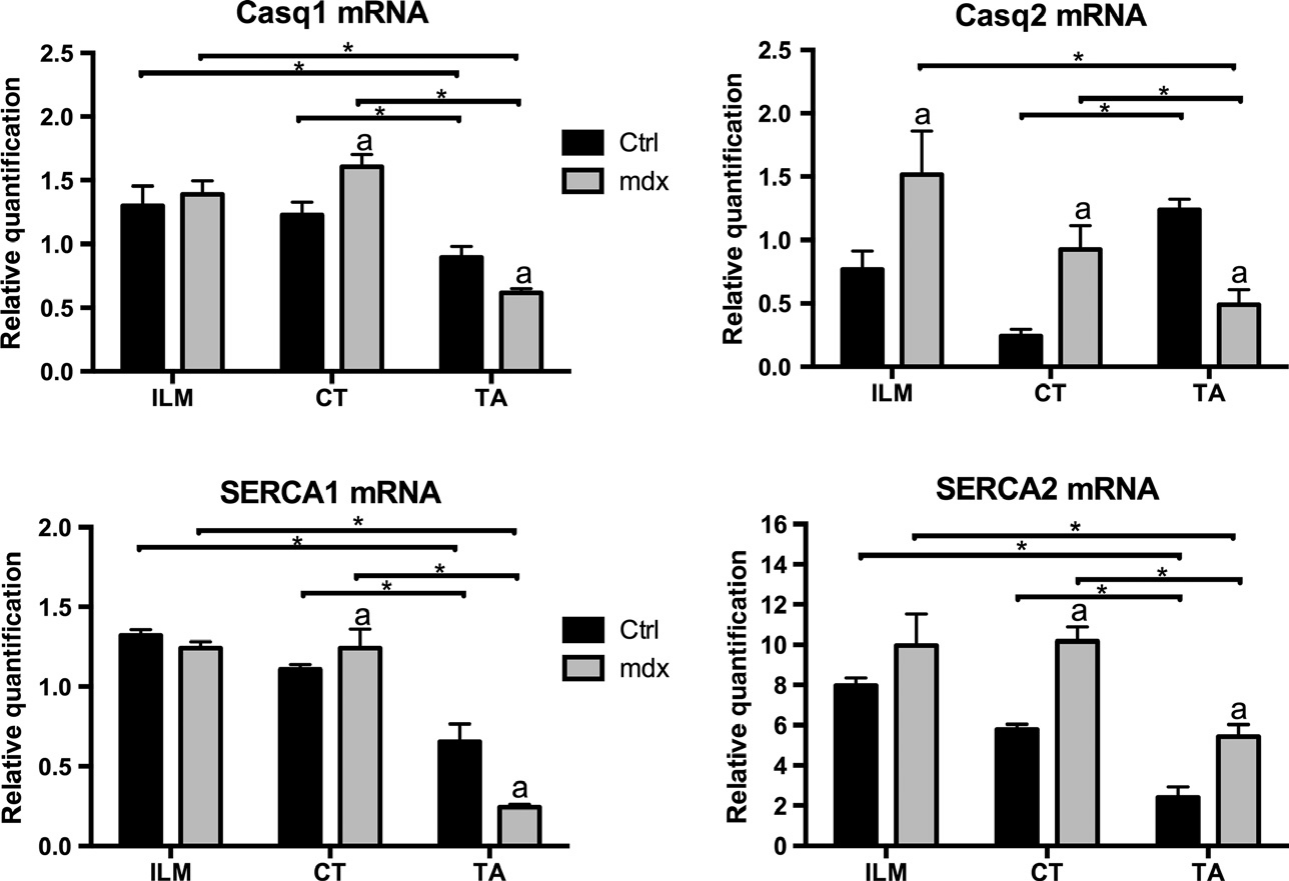
Relative mRNA levels of genes encoding Ca^2+^-handling proteins in control (Ctrl) and dystrophic (*mdx*) mice in intrinsic laryngeal muscles (ILM), cricothyroid (CT) muscle, extraocular muscle (EOM), and *tibialis anterior* (TA) muscle. The genes studied were Ca^2+^-ATPase slow twitch (*Serca2*), fast twitch (*Serca1*), calsequestrin 2 (*Casq2*), and calsequestrin 1 (*Casq1*). The relative mRNA levels of genes encoding Ca^2+^-handling proteins were determined by SYBR Green qPCR in muscle tissue. Mean fold changes ± SEM of three independent samples each were calculated and statistically analyzed using REST 2005. Each independent muscle sample contained a pool of muscles from 3–4 mice. Asterisks indicate statistical significance (*P < 0.05). Letter “a” was considered statistically significant (*P* < 0.05) compared with Ctrl.

The dystrophic ILM showed increased expression of *Casq1, Casq2, Serca1,* and *Serca2* compared to the limb dystrophic muscle (Fig.3). In the *mdx* ILM, gene expression of these SR calcium-related proteins was comparable (*Casq1, Serca1,* and *Serca2*) or increased (*Casq2*) in comparison to control ILM. The gene expression of all the proteins (*Casq1, Casq2, Serca1,* and *Serca2*) was elevated in the affected CT muscle in relation to its respective control.

## Discussion

We found that the Ca^2+^ reuptake-related proteins of the sarcoplasmic reticulum, *Serca1* and *Serca2*, the Serca regulator *Pln* and the Ca^2+^-binding protein *Casq1* and *Casq2* were expressed at higher mRNA levels in rat ILM compared to limb muscle. In addition, plasma membrane calcium pumps (*Pmca* and *Ncx*) were significantly increased in rat ILM compared to TA. These results show that rat ILM myofibers display a Ca^2+^-buffering profile that predict a better ability to handle increases in the cytosolic Ca^2+^, in comparison to the limb muscle, which may help protect them from pathophysiological conditions.

Previously, we and others demonstrated that ILM from the *mdx* mice are spared from dystrophy (Marques et al. 2007; Thomas et al. 2008; Smythe 2009), possibly due to higher levels of the calcium-binding proteins calsequestrin and Serca (Ferretti et al. 2009). In this study, we add further information regarding gene expression of these proteins in dystrophic ILM and found a similar pattern seen in the rat, that is, the expression of these proteins is augmented in ILM (in the dystrophic and in the rat) compared to limb muscle (in the dystrophic and in the rat; except for Serca1, which showed comparable expression in rat ILM x TA). The present results suggest that calsequestrin and SERCA expression and levels (Ferretti et al. 2009) profile is a constitutive feature of laryngeal muscles and may help protect ILM myofibers from Ca^2+^-mediated damage, at least in muscular dystrophy. Furthermore, these results suggest that this profile does not necessarily represent an adaptation to the lack of dystrophin in the *mdx* ILM.

*Cacna1s* gene is a voltage-gated calcium channel that encodes the alpha-1S subunit of the DHPR, which is the conducting channel and carries the voltage sensor- and dihydropyridine-binding site (Krasnyi and Ozerniuk 2011). Cacna1s has been involved in several biological processes and disorders of skeletal muscles, such as malignant hyperthermia and muscle channelopathies (Maclennan and Zvaritch 2011; Burge and Hanna 2012). The RyR1 channel complex is mainly involved in promoting the release of Ca^2+^ from the SR, dampening calcium oscillation that is required for continued myofiber contractions (Bellinger et al. 2008). The abundant SR in ILM (compared to limb muscle) (Hinrichsen and Dulhunty 1982) could implicate in more releasable sites and consequently higher levels of *Cacna1s* and *RyR1* in ILM than in TA muscle. However, these two Ca^2^ -release proteins were expressed in similar levels in rat ILM and TA. One possibility to explain this finding could be related to differences in the activity status of these proteins, rather than their expression, between ILM and TA muscles. Overall, the present findings (i.e., higher levels of SR Ca^2+^-reuptake *x* similar levels of SR Ca^2+^-release proteins in ILM vs. TA) raise the possibility that Ca^2+^-reuptake mechanisms are more important than Ca^2+^-release mechanisms for ILM myofibers to maintain their functional properties, but further studies would be necessary to elucidate this point.

Besides the SR, another Ca^2+^ influx pathway is the store-operated Ca^2+^ entry (SOCE), which is stimulated by reduction in intracellular Ca^2+^ stores (Putney 1990). The molecular components of the SOCE include STIM1, an endoplasmic/sarcoplasmic reticulum (ER/SR) Ca^2+^ sensor (Liou et al. 2005; Zhang et al. 2005) that translocates from the ER/SR membrane to regions close to the plasma membrane following depletion of the intracellular Ca^2+^ stores (Wu et al. 2006). This movement of STIM1 activates Orai1, a pore-forming unit that allows permeation of Ca^2+^ through the plasma membrane into the cytosol (Prakriya et al. 2006; Wang et al. 2010). Orai1 was significantly increased in rat ILM in comparison to TA muscle. Orai1 protein abundance is under regulation of a wide variety of cellular mechanisms, including hormones (Lang et al. 2012). ILM are under the modulation of several hormones that can influence their fiber types (Hoh 2005), which could explain the differences between ILM and TA muscle observed here regarding their Orai1 profile.

In this study, we also explored the levels of the cytoplasmic-buffering proteins Pvalb and CaM. The genes for these proteins were significantly increased in rat ILM compared to TA. It is known that fast contracting fibers, such as ILM myofibers (Hoh 2005), contain high concentrations of Pvalb (Heizmann et al. 1982). However, *CaM* mRNA expression in ILM was almost 10 times higher than *Pvalb* and threefold increased in ILM compared to TA. This finding suggests that, in ILM, calmodulin may represent an important mechanism of cytosolic Ca^2+^ buffering. Furthermore, similarly in EOM, Ca^2+^-CaM signaling mechanisms in ILM may ensure the production of other molecules, such as utrophin, to help protect against dystrophy (Porter et al. 1998).

Cricothyroid (CT), the vocal cord tensor, resembles limb muscles in MyHC composition and fiber-type properties (Hoh 2005). Considering their calcium-buffering profile, we observed that rat CT shares some similarities with the limb muscle (*Serca2, Ncx, Casq2, and Pvalb*), but also shows similarities with others ILM (*Serca1, Casq1, RyR1, CaM, and Orai1*). Previously, using histopathological methods, we demonstrated that the dystrophic CT muscle was affected significantly, showing an increased percentage of central nucleated fibers when compared to the other ILM (Marques et al. 2007). We suggested that decreased expression of calcium-binding proteins could explain the susceptibility of CT to dystrophy (Ferretti et al. 2009). However, in this study, we observed augmented gene expression of all the main SR calcium-related proteins, including *Serca2*, in the *mdx* CT, suggesting that other calcium-related proteins and/or mechanisms may be involved in CT dystrophy. We observed a dramatic increase in *Rgn* in rat CT compared to other ILM and TA. Rgn is an important cytosolic calcium-handling protein, involved in several intracellular signaling mechanisms (Yamaguchi 2005) and may be involved in the functional differences among CT and other ILM previously reported (Hinrichsen and Dulhunty 1982). This study also points out that CT and ILM present a distinct expression profile for wild-type rats and mice regarding *Sercas* and *Casq2*. A possible explanation for this finding could be related to differences in the rate of respiration due to distinct metabolic rates in rats compared to mice or distinct vocal repertoire (Holy and Guo 2005; Riede 2014). Differences in the contraction times of laryngeal and CT muscles among different species have also been reported, possibly due to differences in SR volume and the content of parvalbumin, a Ca^2+^-binding protein (Hoh 2005).

Higher levels of Pln, CaMKII, and CaM in rat ILM may play a role in regulating Serca activity (O'Brien et al. 1993; Morita et al. 2009), through similar mechanisms as those described for the EOM (Simmerman et al. 1986; Traaseth et al. 2008; Zeiger et al. 2010). In this study, we also add information about the SOCE component Orai1 in ILM and EOM. We observed that Orai1 showed a similar profile in ILM versus EOM, that is, significantly elevated compared to the limb muscle. Interestingly, EOM showed significantly higher levels of these proteins in relation to ILM. Overall, despite both muscles groups (ILM and EOM) display very fast contraction cycles, they have minimal differences in their Ca^2+^-buffering profile that may reflect differences in their functional properties, such as the fine control of airway caliber by ILM and fine control of eye movement, by the EOM.

Vocal fatigue, a symptom of a voice disorder that occurs as a consequence of prolonged voice use mainly reported in singing, teaching and acting professionals (Welham and Maclagan 2003), may contribute to the development of other laryngeal pathologies (Kostyk and Rochet 1998). While the etiology of vocal fatigue is poorly understood, neuromuscular junction fatigue, changes in vocal fold viscosity and reduced blood circulation seem to be involved (Welham and Maclagan 2003). Fatigue in skeletal muscles seems to be related to a decrease in the amplitude of the myoplasmic Ca^+2^ transient which is thought to result from changes in the function of SR-calcium-related proteins (Fitts 2011). Recently, the plasma membrane transporter NCX has been suggested to play a role in the resistance to muscle fatigability in slow twitch muscles (Michel et al. 2014). We observed that most (58%) of the calcium-related proteins mRNAs in the rat were significantly lower expressed in the CT muscle in comparison with other ILM, including the NCX transporter. Therefore, although there are no evidences from in vivo studies in humans, it may be that the human CT muscle is less resistant to fatigue compared to other ILM. Overall, it seems reasonable to suggest that alterations in calcium homeostasis due to changes in calcium-related proteins may help explain, at least in part, the decreases in ILM resistance to fatigability in certain pathologies and this may be of relevance to develop new therapeutic strategies to vocal muscle fatigue or even to other laryngeal disturbances.

## Conclusions

This study demonstrates that rat ILM present a constitutive Ca^2+^-buffering profile that predicts their better ability to handle calcium changes in comparison to limb muscles. This profile is in agreement with their function as very fast muscles with a well-developed capacity for prolonged work. Other mechanisms may help explain the differential functional and pathophysiological responses of ILM versus limb muscles. For instance, it would be interesting to study other important SR calcium-related proteins, such as sarcalumenin (Jiao et al., 2012) and sarcolipin (Rossi and Dirksen 2006), which are major Ca^2+^-binding and SERCA regulators (Dowling et al. 2004). Nevertheless, this study suggests that mechanisms involved in the prompt sequestering of Ca^2+^ (sarcoplasmic reticulum Ca^2+^-reuptake proteins, plasma membrane pumps, and cytosolic Ca^2+^-buffering proteins) are particularly elevated in ILM, indicating their importance for ILM myofiber function and protection against disease. Furthermore, differential levels of Orai1 in rat ILM and EOM over the limb muscle suggests a role for SOCE in ILM/EOM functional properties and signaling mechanisms.

## Conflict of Interest

None declared.

## References

[b1] Beard NA, Laver DR, Dulhunty AF (2004). Calsequestrin and the calcium release channel of skeletal and cardiac muscle. Prog. Biophys. Mol. Biol.

[b2] Bellinger AM, Mongillo M, Marks AR (2008). Stressed out: the skeletal muscle ryanodine receptor as a target of stress. J. Clin. Invest.

[b3] Berchtold MW, Brinkmeier H, Muntener M (2000). Calcium ion in skeletal muscle: its crucial role for muscle function, plasticity, and disease. Physiol. Rev.

[b4] Berridge MJ, Bootman MD, Roderick HI (2003). Calcium signalling: dynamics, homeostasis and remodelling. Nat. Rev. Mol. Cell Biol.

[b5] Burge JA, Hanna MG (2012). Novel insights into the pathomechanisms of skeletal muscle channelopathies. Curr. Neurol. Neurosci. Rep.

[b6] Bulfield G, Siller WG, Wight PA, Moore KJ (1984). X chromosome-linked muscular dystrophy (mdx) in the mouse. Proc. Natl Acad. Sci. USA.

[b7] Chin ER (2010). Intracellular Ca^2+^ signaling in skeletal muscle: decoding a complex message. Exerc. Sport Sci. Rev.

[b8] Debain JJ, Lebrigand H, Freyss G (1968). Laryngeal manifestations of myasthenia. Apropos of a case affecting the dilator muscles of the glottis. Ann. Otolaryngol. Chir. Cervicofac.

[b9] DelGaudio JM, Sciote JJ, Carroll WR, Escalmado RM (1995). Atypical myosin heavy chain in rat laryngeal muscle. Ann. Otol. Rhinol. Laryngol.

[b10] Dowling P, Doran P, Ohlendieck K (2004). Drastic reduction of sarcalumenin in Dp427 (dystrophin of 427 kDa) – deficient fibres indicates that abnormal calcium handling plays a key role in muscular dystrophy. Biochem. J.

[b11] Feng X, Zhang T, Ralston TE, Ludlow CL (2012). Differences in neuromuscular junctions of laryngeal and limb muscles in rats. Laryngoscope.

[b12] Ferretti R, Marques MJ, Pertille A, Santo Neto H (2009). Sarcoplasmic-endoplasmic-reticulum Ca^2+^-ATPase and calsequestrin are overexpressed in spared intrinsic laryngeal muscles of dystrophin-deficient mdx mice. Muscle Nerve.

[b13] Fitts RH (2011). New insights on sarcoplasmic reticulum calcium regulation in muscle fatigue. J. Appl. Physiol.

[b15] Gailly P (2002). New aspects of calcium signaling in skeletal muscle cells: implications in Duchenne muscular dystrophy. Biochim. Biophys. Acta.

[b16] Goding GS, Al-Sharif KI, Mc Loon LK (2005). Myonuclear addition to uninjured laryngeal myofibers in adult rabbits. Ann. Otol. Rhinol. Laryngol.

[b17] Heizmann CW, Berchtold MW, Rowlerson AM (1982). Correlation of parvalbumin concentration with relaxation speed in mammalian muscles. Proc. Natl Acad. Sci.

[b18] Hinrichsen C, Dulhunty A (1982). The contractile properties, histochemistry, ultrastructure and electrophysiology of the cricothyroid and posterior cricoarytenoid muscles in the rat. J. Muscle Res. Cell Motil.

[b19] Holy TE, Guo Z (2005). Ultrasonic songs of male mice. PLoS Biol.

[b20] Hoh JF (2005). Laryngeal muscle fibre types. Acta Physiol. Scand.

[b21] Jiao Q, Takeshima H, Ishikawa Y, Minamisawa S (2012). Sarcalumenin plays a critical role in age-related cardiac dysfunction due to decreases in SERCA2a expression and activity. Cell Calcium.

[b22] Kersing W, Jennekens FG (2004). Age-related changes in human thyroarytenoid muscles: a histological and histochemical study. Eur. Arch. Otorhinolaryngol.

[b23] Kostyk BE, Rochet AP (1998). Laryngeal airway resistance in teachers with vocal fatigue: a preliminary study. J. Voice.

[b24] Krasnyi AM, Ozerniuk ND (2011). The expression of genes encoding the voltage-dependent L-type Ca^2+^ channels in proliferating and differentiating C2C12 myoblasts of mice. Izv. Akad. Nauk Ser. Biol.

[b25] Khurana TS, Prendergast RA, Alameddine HS, Tome FM, Fardeau M, Arahata K (1995). Absence of extraocular muscle pathology in Duchenne's muscular dystrophy: role for calcium homeostasis in extraocular muscle sparing. J. Exp. Med.

[b26] Lang F, Eylenstein A, Shumilina E (2012). Regulation of Orai1/STIM1 by the kinases SGK1 and AMPK. Cell Calcium.

[b27] Li M, Chen S, Wang W, Chen D, Zhu M, Liu F (2014). Effect of duration of denervation on outcomes of ansa-recurrent laryngeal nerve reinnervation. Laryngoscope.

[b28] Liou J, Kim ML, Heo WD, Jones JT, Myers JW, Ferrell JE (2005). STIM is a Ca^2+^ sensor essential for Ca^2+^-store-depletion-triggered Ca^2+^ influx. Curr. Biol.

[b29] Maclennan DH, Zvaritch E (2011). Mechanistic models for muscle diseases and disorders originating in the sarcoplasmic reticulum. Biochim. Biophys. Acta.

[b30] Maranillo E, Leon X, Orus C, Quer M, Sanudo JR (2005). Variability in nerve patterns of the adductor muscle group supplied by the recurrent laryngeal nerve. Laryngoscope.

[b31] Marques MJ, Ferretti R, Vomero VU, Minatel E, Neto HS (2007). Intrinsic laryngeal muscles are spared from myonecrosis in the mdx mouse model of Duchenne muscular dystrophy. Muscle Nerve.

[b32] Mao VH, Abaza M, Spiegel JR, Mandel S, Hawkshaw M, Heuer RJ (2001). Laryngeal myasthenia gravis: report of 40 cases. J. Voice.

[b33] McLoon LK, Thorstenson KM, Solomon A, Lewis MP (2007). Myogenic precursor cells in craniofacial muscles. Oral Dis.

[b34] Melzer W, Herrmann-Frank A, Luttgau HC (1995). The role of Ca^2+^ ions in excitation-contraction coupling of skeletal muscle fibres. Biochim. Biophys. Acta.

[b35] Michel LYM, Verkaart S, Koopman WJH, Willems PHGM, Hoenderop JGJ, Bindels RJM (2014). Function and regulation of the Na^+^-Ca^2+^ exchanger NCX3 splice variants in brain and skeletal muscle. J. Biol. Chem.

[b36] Morita M, Iguchi A, Takemura A (2009). Roles of calmodulin and calcium/calmodulin-dependent protein kinase in flagellar motility regulation in the coral Acropora digitifera. Mar. Biotechnol.

[b37] Nixon I, Ramsay S, MacKenzie K (2010). Vocal function following discharge from intensive care. J. Laryngol. Otol.

[b38] O'Brien J, Meissner G, Block BA (1993). The fastest contracting muscles of nonmammalian vertebrates express only one isoform of the ryanodine receptor. Biophys. J.

[b39] Porter JD, Rafael JA, Ragusa RJ, Brueckner KB, Trickett JI, Davies KE (1998). The sparing of extraocular muscle in dystrophinopathy is lost in mice lacking utrophin and dystrophin. J. Cell Sci.

[b40] Prakriya M, Feske S, Gwack Y, Srikanth S, Rao A, Hogan PG (2006). Orai1 is an essential pore subunit of the CRAC channel. Nature.

[b41] Putney JW (1990). Capacitative calcium entry revisited. Cell Calcium.

[b42] Riede T (2014). Rat ultrasonic vocalization shows features of a modular behavior. J. Neurosci.

[b43] Rosenthal LHS, Benninger MS, Deeb RH (2007). Vocal fold immobility: a longitudinal analysis of etiology over 20 years. Laryngoscope.

[b44] Rossi AE, Dirksen RT (2006). Sarcoplasmic reticulum: the dynamic calcium governor of muscle. Muscle Nerve.

[b45] Simmerman HK, Collins JH, Theibert JL, Wegener AD, Jones LR (1986). Sequence analysis of phospholamban. Identification of phosphorylation sites and two major structural domains. J. Biol. Chem.

[b46] Shinners MJ, Goding GS, McLoon LK (2006). Effect of recurrent laryngeal nerve section on the laryngeal muscles of adult rabbits. Otolaryngol. Head Neck Surg.

[b47] Smythe GM (2009). Dystrophic pathology in the intrinsic and extrinsic laryngeal muscles in the mdx mouse. J. Otolaryngol. Head Neck Surg.

[b48] Traaseth NJ, Ha KN, Verardi R, Shi L, Buffy JJ, Masterson LR (2008). Structural and dynamic basis of phospholamban and sarcolipin inhibition of Ca(2+)-ATPase. Biochemistry.

[b49] Thomas LB, Joseph GL, Adkins TD, Andrade FH, Stemple JC (2008). Laryngeal muscles are spared in the dystrophin deficient mdx mouse. J. Speech. Lang. Hear. Res.

[b50] Wang Y, Deng X, Mancarella S, Hendron E, Eguchi S, Soboloff J (2010). The calcium store sensor, STIM1, reciprocally controls Orai and CaV1.2 channels. Science.

[b51] Welham NV, Maclagan MA (2003). Vocal fatigue: current knowledge and future directions. J. Voice.

[b52] Wu MM, Buchanan J, Luik RM, Lewis RS (2006). Ca^2+^ store depletion causes STIM1 to accumulate in ER regions closely associated with the plasma membrane. J. Cell Biol.

[b53] Xu W, Zhao G, Hu H, Fan E (2009). Characteristics of intrinsic laryngeal muscle after recurrent laryngeal nerve injury. Lin. Chung. Er. Bi. Yan. Hou. Tou. Jing. Wai. Ke. Za. Zhi.

[b54] Yamaguchi M (2005). Role of regucalcin in maintaining cell homeostasis and function. Int. J. Mol. Med.

[b55] Zeiger U, Mitchell CH, Khurana TS (2010). Superior calcium homeostasis of extraocular muscles. Exp. Eye Res.

[b56] Zhang SL, Yu Y, Roos J, Kozak JA, Deerinck TJ, Ellisman MH (2005). STIM1 is a Ca^2+^ sensor that activates CRAC channels and migrates from the Ca^2+^ store to the plasma membrane. Nature.

